# The complete mitochondrial genome of the Japanese honeybee, *Apis cerana japonica* (Insecta: Hymenoptera: Apidae)

**DOI:** 10.1080/23802359.2016.1144108

**Published:** 2016-02-10

**Authors:** Jun-ichi Takahashi, Takeshi Wakamiya, Takuya Kiyoshi, Hironobu Uchiyama, Shunsuke Yajima, Kiyoshi Kimura, Tetsuro Nomura

**Affiliations:** a Department of Life Sciences, Kyoto Sangyo University, Kyoto, Japan;; b Department of Zoology, National Museum of Nature and Science, Tokyo, Japan;; c NODAI Genome Research Center, Tokyo University of Agriculture, Tokyo, Japan;; d Department of Bioscience, Tokyo University of Agriculture, Tokyo, Japan;; e Honeybee Research Group, Animal Breeding and Reproduction Research Team, National Institute of Livestock and Grassland Science, Tsukuba, Japan

**Keywords:** *Apis cerana*, Asian honeybee, genetic distance, Illumina sequencing, subspecies

## Abstract

In this study, we analyzed the complete mitochondrial genome of the Japanese honeybee *Apis cerana japonica*. The mitochondrial genome of *A. c. japonica* is a circular molecule of 15 917 bp and is similar to that of *A. c. cerana*. It contains 13 protein-coding genes, 22 tRNA genes, two rRNA genes and one A + T-rich control region. All protein-coding genes are initiated by ATT and ATG codons and are terminated by the typical stop codon TAA or TAG, except for the start codon of *ATP8* which ends with C. All tRNA genes typically form a cloverleaf secondary structure, except for *tRNA*-*Ser* (*AGN*).

Asian hive bees from the *Apis cerana* group (comprising four or more subspecies and three related species) are of particular interest because they are widely distributed across the Asian continent and the islands, showing interesting patterns of intra- and inter-species differences caused by repeated isolation and re-unification of populations due to repeated changes in sea levels (Oldroyd & Wongsiri 2006). Here, we reported the complete mitochondrial genome of the Japanese honeybee *A. c. japonica* (accession number AP017314) which will be useful for various studies in the *A. cerana* group.

The adult worker was collected from a hive of an apiary of Kyoto Sangyo University (KSU) in Kyoto, Japan (specimen is stored in the KSU, number ACJ007). Genomic DNA isolated from males was sequenced using Illumina’s HiSeq platform (Illumina). The resultant reads were assembled and annotated using the MITOS web server (Bernt et al. 2013, Germany) and GENETYX v.10 (Genetyx, Japan). The phylogenetic analysis was constructed using the MEGA6 (Tamura et al. 2013) based on the nucleotide sequences of the 13 protein-coding genes.

The *A. c. japonica* mitochondrial genome forms a closed loop that is 15 917 bp long. The *A. c. japonica* mitochondrial genome represents a typical hymenopteran mitochondrial genome molecule and matches the common *A. c. cerana* organization in that it comprise 13 protein-coding genes, 22 putative tRNA genes, two rRNA genes and an A + T-rich control region. The average AT content of the *A. c. japonica* mitochondrial genome was 83.4%. Similar to honeybee mitochondrial genomes, the heavy strand encodes nine protein-coding genes and 14 tRNA genes. The light strand encodes four protein-coding genes, eight tRNAs and two rRNA genes. The *ATP6* and *ATP8* genes shared 19 nucleotides, and the *ND4* and *ND4L* shared five nucleotides. Nine protein-coding genes of the *A. c. japonica* mitochondrial genome started with ATT, the *ATP6*, *COIII* and *Cytb* genes with ATG, and the *ATP8* gene with ATC, all of which have been commonly found in the *A. c. cerana* mitochondrial genome (Tan et al. 2011). The stop codon of each of these protein-coding genes was either TAA or TAG, similar to the case in other honeybees. All of the tRNA genes typically possessed cloverleaf secondary structures, except for *tRNA-Ser* (*AGN*), which lacks a dihydrouridine (DHU) arm.

Phylogenetic analysis was constructed by applying 13 mitochondrial protein-coding genes with 13 closely related taxa (Figure 1). The genetic distance of the *A. cerana* subspecies mitochondrial genome was 0.006. This corresponds well to the genetic distance generally observed in four *A. mellifera* subspecies mitochondrial genomes (Crozier & Crozier 1993; Gibson & Hunt 2015; Haddad 2015; Hu et al. 2015), which range from 0.004 in African subspecies to 0.014 in European-Middle Eastern subspecies. The 88 mutation sites between *A. c. japonica* and *A. c. cerana* were evenly distributed over the mitochondrial genome, except for the A + T-rich control region. The complete sequence of the *A. cerana* mitochondrial genome provides additional genetic tools for studying population genetics, speciation and biogeography of this group.

**Figure 1.**
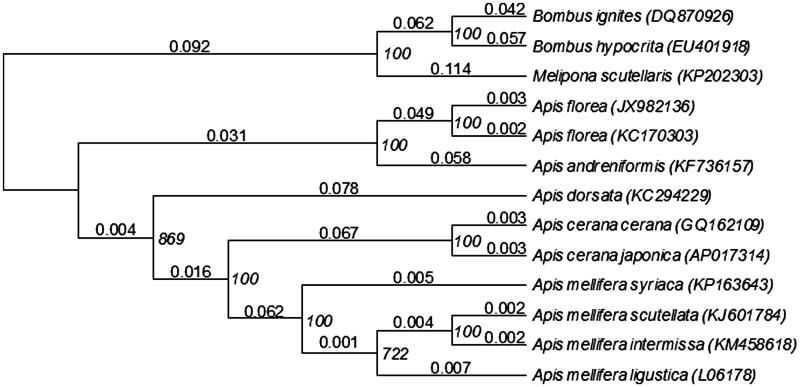
Phylogenetic relationships (neighbour-joining) of the genus Apis of Hymenoptera based on the mitochondrial genome nucleotide sequence of the 13 protein-coding genes. Numbers on each node indicate the genetic distances supported in an analysis of Tamura–Nei model. Numbers (Italic) beside nodes are per cent of 1000 bootstrap values. The Bombus ignites, B. hypocrita and Melipona scutellaris were used as an outgroup. Alphanumeric in parentheses indicates the GenBank accession numbers.
